# Self-rated health, work characteristics and health related behaviours among nurses in Greece: a cross sectional study

**DOI:** 10.1186/1472-6955-4-8

**Published:** 2005-12-20

**Authors:** Noula A Pappas, Yannis Alamanos, Ioannis DK Dimoliatis

**Affiliations:** 1 Department of Hygiene and Epidemiology, University of Ioannina School of Medicine, Ioannina, 45110, Greece

## Abstract

**Background:**

Previous studies on self-rated health among nurses have indicated an association of low job satisfaction and stress in relation to poor self-rated health. The relationship between self rated health and the specific work characteristics and health related behaviours of nurses to our knowledge have not been adequately studied.

**Objective:**

To investigate the health profile of nurses working in hospitals in North West Greece and to examine the associations between self rated health (SRH) and health related behaviours and work characteristics in this group of hospital employees.

**Methods:**

A self-administered questionnaire was distributed to a random sample of 443 nurses working in all the hospitals in North West Greece. Regression analysis was used to examine the relationship of health related behaviours and work characteristics with self rated health among the nurses.

**Results:**

A total of 353 responded to the questionnaire (response rate 80%) of which 311 (88%) were female and 42 (12%) male. The mean age (standard deviation) of the respondents was 36 years (5.6) and their mean years of working as nurses were 13.5 years (5.9). Almost half of the nurses' smoked, and about one third were overweight or obese. About 58% (206) of the nurses reported having poor health while 42% (147) reported having good health. Self-rated health was independently associated with gender, effort to avoid fatty foods and physical activity, according to multiple logistic regression analysis.

**Conclusion:**

The population studied presented a relatively poor health profile, and a high proportion of poor SRH. Though female gender and effort to avoid fatty foods were associated with poor SRH, and exercise and white meat consumption with good SRH, specific work characteristics were not associated with SRH.

## Background

Self-rated health (SRH) has become one of the most common health indicators in Public Health research. Prospective and cross sectional studies have shown that a persons' own perception of their general health is a powerful predictor of subsequent mortality [1-3], incorporates psychological well being and social functioning [4-8], while cross sectional studies indicate that SRH is a significant predictor of health care utilization [9,10].

Health behaviours such as smoking, alcohol consumption and leisure time exercise are associated with SRH [6,11,12]. When investigating the effect of health behaviours on self-rated health, it has been suggested that inactive and smoking persons have an increased risk for a decline in health status, compared to active and non-smoking persons [13]. Moderate alcohol intake is associated with good ratings of health [14], and self-rated health among sport participants is considered to be better than that of non-participants [15,16].

People tend to assign differential value to various factors when assessing their health status; they do not use the same frame of reference. The elderly are more likely to consider limitations in their functioning abilities [17] or even physical symptoms rather than manifest disease [18], while young people take their health behaviours into account when rating their health status [19]. The specific referents used may vary by age, education or "racial" ancestry [19,20], and when sufficient and detailed medical information is available, socio-cultural factors may contribute only marginally to self- rating of health [17]. Furthermore evidence concerning especially socioeconomic or "racial" ancestry differences in the way health ratings are made is not fully consistent across studies.

Nurses constitute a group of employees presenting a specific interest in health perception and health related behaviours, because of their professional involvement with these issues and because of their vital role in health promotion [21].

Prospective studies show that high psychological and physical demands, low decision authority and low social support in the work environment are predictive of poor self-rated health [22,23]. Similarly, cross sectional studies seem to find a relationship between poor SRH and low job satisfaction [24,25], increased workload and burnout [24,26], and lack of peer support in their professional environment [27]. For example nurse managers exposed to high job demands had significantly increased odds for poor self-rated health [27] and report high levels of burnout [24]. Besides stress, psychological load and strain may have a cumulative negative effect on general and mental health in female personnel working in health care [28].

Although a lot of research has been conducted on low job satisfaction and stress in relation to poor SRH among nurses [21,29-31], the relationship between health related behaviours and work characteristics, such as staff position (nurse manager, ward nurse), or department of work (internal medicine, surgery etc.), shift work, to our knowledge, has not been adequately studied. We thus designed and conducted a cross sectional survey among nurses working in hospitals in North West Greece in order to study their health profile and to assess the effect of work characteristics and health related behaviours on their self- rating of health.

## Methods

### Sample

The study was conducted in all seven hospitals of the North West Region of Greece, which at the time employed 1329 nurses. The study area represents a population of 488,435 inhabitants according to the National Census of 2001, including four districts situated on the mainland and two districts situated in islands. Urban residents represented about one third of the total population, living in the capitals of the districts. The study area presents significant socio-economic variations, including some areas with the lowest income per capita in the European Union.

The selection criterion was working as a nurse in one of the seven hospitals of the selected region. Using the human resources department files of each hospital, one third of the nursing population working in each department was randomly selected to participate in this study, with the use of random numbers. Nurses on long-term sick leave or maternity leave were excluded from the study after obtaining these files. A total of 443 nurses were included in the sample.

A written explanation of the study was provided to all the participants. The participants were given a self-administered questionnaire in May 2002. The non-responders were reminded with a written letter and a telephone call at their place of work.

### Questionnaire

The questionnaire included questions about sociodemographic factors, work characteristics, health related behaviours and SRH.

The sociodemographic factors that were included in the questionnaire were gender, age, marital status (single, married, divorced, widowed) and educational level (two, three, or four year nursing degree).

Work characteristics included questions about position (head nurse, charge nurse, and staff nurse), rotating shift work (average number of night and evening shifts a week), total years working as a nurse, and department of employment. Department of employment was classified into four categories: internal medicine, surgical, intradepartmental and administration. The category of internal medicine included departments such as internal medicine, paediatrics, dermatology, neurology, and cardiology. The surgical category included surgical department, neurosurgery, orthopaedic, heart surgery, urology and ophthalmology departments. Intradepartmental category included the emergency room, out patient unit, intensive care unit and the operating room.

Health related behaviours included questions about smoking (current, former, never smoker), alcohol consumption (never drinking, occasional drinking, drinking once a week, drinking more than once a week), physical activity (exercising vs. not exercising), dietary habits (weekly white and red meat consumption, effort to avoid fat in their daily diet, and fibre consumption), and average sleep duration (average hours of daily sleep). Body mass index (BMI) was estimated on self reported weight and height.

Self-rated health was assessed on a five-point scale, ranging from excellent to poor (excellent, good, fair, poor, and very poor), through the following question: "In general how would you say your health is?" This is a single item measure and as such its reliability and validity was established in previous studies [32].

Two pilot studies were conducted in order to clarify any misunderstandings of the questions included in the questionnaire. Upon collection, revisions were made to simplify and improve the questionnaire. Each questionnaire was coded to ensure confidentiality of the participant responses.

### Statistical analysis

Differences in the characteristics of the respondents with poor health and good health were tested accordingly using chi-square for categorical variables and t-test for continuous variables.

Logistic regression was performed using SRH as the dependent variable, to assess the associations between SRH and other parameters. Self-rated health was dichotomized and was coded 0 for respondents reporting excellent or good (herein referred to as good health) and 1 for those reporting fair, poor or very poor health (herein referred to as poor health). Categorical predictor variables with more than two categories were appropriately coded using dichotomous dummy variables.

First, associations of SRH with work characteristics and health related behaviours were considered using univariate models. All associations of SRH with health related behaviours and work characteristics that were found to be statistically significant in the univariate analysis were introduced in the multivariate model. Additional sensitivity analyses considered men and women separately. However, since only 42 men were included in our sample, to avoid overfitting, multivariate analysis was considered not appropriate.

Analysis was conducted in SPSS 10.0 (SPSS Inc. Chicago, IL). A p-value <0.05 indicates a statistically significant result. All p-values are two-tailed.

## Results

A total of 443 nurses were included in the sample. A total of 353 responded to the questionnaire (response rate 80%) of which 311 (88%) were female and 42 (12%) male.

Seventy-four (93%) of the non-respondents were female and six (7%) male; there was no statistically significant difference between respondents and non-respondents according to gender (p > 0.1). Almost 70% of the non-respondents were nurses working in the two large hospitals of the selected Region, while 22% were nurses working in a hospital located on the island Corfu; the difference between the respondents and non-respondents according to hospital was statistically significant (p < 0.001).

The mean age of the respondents was 36 years and their mean years of working as nurses were 13.5 years. Almost half (47%) of the participants had a two-year nursing degree, while 53% had a three or four year nursing degree. The majority of the respondents were staff nurses (79%) as opposed to 21% that were charge nurses and nurse managers. About 40% of the nurses worked in an intradepartmental unit, while 31% worked in an internal medicine department, 24% in a surgery department, and only five percent worked in administration. Thirty six (10.2%) nurses reported having excellent health, 111 (31.4%) good health, 136 (38.5%) fair health, 64 (18.1%) poor health and six (1.7%) very poor health.

Characteristics of the 206 nurses who reported having poor health are shown in table 1 in comparison with nurses who reported having good health. Nurses with poor SRH were significantly more likely to be female, between the ages of 30–39 years old, married, have 11–20 years working as a nurse, and work in rotating shifts.

**Table 1:** Characteristics of nurses with good health vs nurses with poor health.

**Variable**	**Frequency n (%)**	**Frequency n (%)**	P value*
			
	**Good health**	**Poor health**	
Total	147	206	
Gender			
Male	30 (20)	12 (6)	<0.001
Female	117 (80)	194 (94)	
Age group			
19–29	19 (13)	12 (6)	<0.001
30–39	108 (74)	135 (67)	
40 and over	20 (14)	56 (28)	
Marital status			
Married	105 (71)	174 (84)	<0.01
Not married	42 (29)	32 (16)	
Education level			
3 or 4 year college degree	84 (58)	101 (49)	NS
2 year college degree	62 (42)	104 (51)	
Department currently working			
Internal Medicine	39 (27)	68 (33)	NS
Surgical department	43 (30)	41 (20)	
Intradepartmental units	60 (41)	82 (40)	
Administration	4 (3)	13 (6)	
Position			
Staff nurse	118 (80)	159 (78)	NS
Charge nurse	14 (10)	18 (9)	
Nurse Manager	15 (10)	28 (14)	
Years working			
<10 years	51 (35)	45 (22)	<0.05
11–20 years	81 (55)	130 (64)	
>20 years	15 (10)	29 (14)	
Shift work			
No	21 (15)	50 (26)	<0.05
Yes	120 (85)	145 (74)	

*P values calculated with *Χ*^2 ^tests, NS = non significant

Health related behaviours among nurses reporting poor health compared to those reporting good health are presented in table 2. Nurses reporting good health were significantly more likely to engage in leisure time exercise, while there was no difference according to smoking, alcohol and BMI. Almost half of the nurses smoked (47%), while only 13% consume alcohol more than once a week. More than one third (36%) of the respondents are overweight or obese. The majority (85%) of the respondents make efforts to include fibre in daily diet and 78% to avoid fatty foods. The mean duration of sleep is about seven hours, ranging from three to ten hours a day.

**Table 2:** Health related behaviours of nurses with good health vs nurses with poor health

**Variable**	**Frequency n (%)**	**Frequency n (%)**	P value*
			
	**Good health**	**Poor health**	
Smoking			
Current smokers	61 (42)	103 (51)	NS
Former smokers	21 (14)	22 (11)	
Have never smoked	65 (44)	79 (39)	
Alcohol consumption			
Does not drink	44 (30)	57 (28)	NS
Occasionally	57 (39)	95 (46)	
Once a week	26 (18)	29 (14)	
More than once a week	20 (14)	25 (12)	
Body Mass Index (BMI)			
<20	12 (8)	16 (8)	NS
20–24.9	89 (61)	104(52)	
25–29.9	36 (25)	67 (33)	
>30	9 (6)	14 (7)	
Leisure time exercise			
Yes	91 (62)	83 (41)	<0.001
No	56 (38)	122 (60)	

*P values calculated with *Χ*^2 ^tests.

Table 3 shows the odds of poor self-rated health and health related behaviours and work related characteristics in univariate and multivariate analysis. In univariate analysis the odds of reporting poor health was four times higher for female nurses, nine percent higher for every one year increase in age, two times higher for married nurses, two times higher for effort to avoid fatty foods, and nine percent higher for every one year working as a nurse. Conversely, exercise and shift work showed about a two-fold improvement in the rating of health, white meat consumption during a week and working in a surgical department showed an increase in the rating of health about one and a half times, and increase in the average hours of sleep showed an improved rating of health 27% for every one-hour increase in sleep.

**Table 3:** Univariate and multivariate associations between poor self-rated health, sociodemographic characteristics, health related behaviours and work related characteristics

		Univariate	Multivariate§
Independent variable		OR (CI 95%)	OR (CI 95%)

Gender	Male	1	1
	Female	4.14 (2.04 to 8.41) ‡	2.72 (1.16 to 6.38) *
Age	per year	1.09 (1.04 to 1.13) ‡	1.10 (0.99 to 1.22)
BMI	per kg/m^2^	1.04 (0.98 to 1.09)	
Marital status	Not married	1	1
	Married	2.18 (1.29 to 3.66) †	1.08 (0.54 to 2.11)
Education	2 year degree	1	
	3 or 4 year degree	0.72 (0.47 to 1.10)	
Exercise		0.42 (0.27 to 0.65) ‡	0.28 (0.16 to 0.48) ‡
Smoking	Currently Smoking	1	
	Quit Smoking	0.62 (0.32 to 1.22)	
	Never smoked	0.72 (0.45 to 1.14)	
Alcohol consumption	Never	1	
	More than once a week	0.96 (0.48 to 1.96)	
	Once a week	0.86 (0.44 to 1.66)	
	Occasionally	1.28 (0.77 to 2.15)	
Red meat consumption		0.94 (0.76 to 1.15)	
White meat consumption		0.66 (0.49 to 0.88) †	0.63 (0.45 to 0.88) †
Effort to avoid fatty foods		2.23 (1.34 to 3.71) †	2.31 (1.23 to 4.37) †
Effort to include fibre in diet		1.14 (0.64 to 2.04)	
Average Sleep duration	Per hour	0.73 (0.61 to 0.88) ‡	0.83 (0.67 to 1.02)
Department	Administration	1	1
	Intradepartmental	0.42 (0.13 to 1.35)	0.62 (0.17 to 2.29)
	Internal Medicine	0.54 (0.16 to 1.76)	1.03 (0.26 to 4.00)
	Surgical	0.29 (0.08 to 0.97) *	0.59 (0.15 to 2.36)
Position	Nurse Manager	1	
	Staff nurse	0.72 (0.37 to 1.41)	
	Charge nurse	0.69 (0.27 to 1.76)	
Total years working	Per year	1.09 (1.05 to 1.13) ‡	0.99 (0.91 to 1.08)
Shift work		0.51 (0.29 to 0.89) †	1.18 (0.56 to 2.50)

OR = odds ratio, CI = confidence interval

* p < 0.05, † p < 0.01, ‡ p < 0.001

§Also adjusted for hospital (not shown)

In multivariate modelling (Table 3) the odds of having poor health increased about three fold for female nurses and two fold for nurses making an effort to avoid fatty foods in their daily diet. In addition exercise proved to be protective, lowering the odds more than three fold for reporting poor health. While statistically significant in univariate modelling, age, marital status, adequate sleep, working in a surgical department, years of working and shift work were eliminated in the multivariate model after adjusting for other variables.

Inferences from analysis as shown in table 4 restricted to women population were very similar to the pooled analysis. Data on men were very few to draw solid conclusions, but qualitatively point to the same direction.

**Table 4:** Univariate and Multivariate associations between poor self-rated health, sociodemographic characteristics, health related behaviours and work related characteristics in men and women

Independent variable		WOMEN		MEN§
		Univariate Analysis	Multivariate Analysis	Univariate
		OR (CI 95%)	OR (CI 95%)	OR (CI 95%)

Age	per year	1.07 (1.03 to 1.13)‡	1.04 (0.94 to 1.16)	1.36 (1.05 to 1.75)*
BMI	per kg/m^2^	1.10 (1.03 to 1.18)†	1.07 (0.98 to 1.17)	1.03 (0.88 to 1.19)
Marital status	Not married	1	1	1
	Married	2.24 (1.27 to 3.93)†	1.35 (0.67 to 2.73)	1.29 (0.28 to 5.89)
Education	2 year degree	1		1
	3 or 4 year degree	0.76 (0.47 to 1.20)		0.26 (0.06 to 1.14)
Exercise		0.42 (0.26 to 0.67)*	0.38 (0.22 to 0.67)†	0.70 (0.18 to 2.77)
Smoking	Currently Smoking	1	1	1
	Quit Smoking	0.47 (0.23 to 0.99)*	0.50 (0.21 to 1.18)	3.40 (0.52 to 22.41)
	Never smoked	0.61 (0.37 to 1.00)	0.56 (0.32 to 0.99)*	1.36 (0.29 to 6.28)
Alcohol consumption	Never	1		1
	More than once a week	2.59 (0.96 to 7.03)		0.24 (0.30 to 1.87)
	Once a week	1.15 (0.56 to 2.33)		0.95 (0.01 to 1.49)
	Occasionally	1.43 (0.84 to 2.43)		0.28 (0.30 to 2.69)
Red meat consumption		1.00 (0.81 to 1.26		0.61 (0.28 to 1.35)
White meat consumption		0.65 (0.47 to 0.88)†	0.66 (0.46 to 0.94)*	0.57 (0.23 to 1.40)
Effort to avoid fatty foods		2.07 (1.18 to 3.61)†	2.43 (1.24 to 4.77)†	2.00 (0.45 to 8.94)
Effort to include fibre in diet		1.07 (0.56 to 2.04)		1.25 (0.22 to 7.28)
Average Sleep duration	Per hour	0.73 (0.61 to 0.88)†	0.79 (0.65 to 0.98)*	0.77 (0.35 to 1.71)
Department	Administration	1		1
	Intradepartmental	0.50 (0.15 to 1.64)		1.92 (0.18 to 20.81)
	Internal Medicine	0.35 (0.10 to 1.18)		
	Surgical	0.61 (0.18 to 2.00)		2.50 (0.24 to 26.47)
Position	Nurse Manager	1		
	Staff nurse	0.79 (0.40 to 1.59)		
	Charge nurse	0.82 (0.31 to 2.19)		
Total years working	Per year	1.07 (1.03 to 1.11)†	1.00 (0.91 to 1.09)	1.44 (1.08 to 1.93)†
Shift work		0.59 (0.34 to 1.02)		

OR = odds ratio, CI = confidence interval

* p < 0.05, † p < 0.01, ‡ p < 0.001

§Since only 42 men were included in our sample, to avoid overfitting, multivariate analysis was considered not appropriate.

## Discussion

The data from this study suggest that Greek nurses present a particular health profile. They often smoke and tend to be overweight compared to the WHO estimates of the general population. There are indications that SRH is influenced by some health behaviours like exercise, effort to avoid fatty foods, etc., rather than work conditions. There is no data on which factors are considered directly when Greek population assesses their own health. Furthermore there is no other Greek study concerning self- rated health among nurses in particular.

We observed a large difference between men and women both in the univariate and multivariate analyses after adjusting for age and other predictors. One possible explanation may be that this is a chance finding due to the small number of male nurses in the sample. The smaller number of male nurses is not a result of biased sampling, since typically male nurses are few in Greece. Other explanations for this difference can also be speculated. It has been suggested that Greeks in general and Greek women in particular express their symptoms more openly than people from other countries. (Although there is no hard evidence to support this) [33]. This is in accordance with previous observations in which women, especially younger women, tend to rate their health as significantly poorer than men do [34,35].

We also observed that Greek nurses tend to smoke more than the general population, which may be attributed to the fact that there is no stringent tobacco control programme in Greek hospitals. According to WHO statistics the prevalence of smoking among the Greek adult population is 38% (female 30%, male 47%). The smoking practices among nurses reported in this study are similar with studies on UK and Italian nurses [36,37]. However Greek nurses seem to smoke more than nurses from the Netherlands, Australia, Israel, Ireland and Hong Kong [38-41].

Alcohol consumption reported by Greek nurses seems to be lower than that of previous studies among nurses in the UK, USA, Ireland [36,41,42]. Compared to the BMI of the general population in Greece [43,44], our findings show that in general Greek nurses are overweight, especially male nurses. The mean body mass index of our sample is higher than that of nurses in other European and Asian countries [36,45]. There is evidence that the rate of obesity in Greece has increased in the last decades and this may be attributed to considerable changes in nutrition and adoption of sedentary lifestyles [48]. It is possible that cultural variations may contribute significantly to differences the above mentioned health related behaviours among Greek nurses and other nurses of other nationalities.

The high proportion of smokers and overweight nurses, the relatively low level of regular physical activity, as well as the low level of alcohol consumption, and the relatively healthy dietary habits found among the Greek nurses may reflect the respective trends in the general Greek population.

The present study shows that health related behaviours such as smoking and alcohol consumption were not associated with self-rated health, whereas effort to avoid fatty foods in daily diet significantly increased the odds of reporting poor health. Conversely, exercise, and white meat consumption were associated with good self-rated health. Work characteristics of the nursing population such as rotating shift work, department of employment, total years of employment and position was not associated with self-rated health after adjusting for sociodemographic factors and health related behaviours.

Previous studies showed that risk factors such as smoking, excessive alcohol consumption, and high body mass index were significantly associated with poor SRH, whereas these factors were not statistically significant in our analysis. This may imply that these health behaviours were not considered directly during the reporting of self-rated health. Another possibility may be the relatively sample size of this study which may be underpowered to detect small differences.

Our findings concerning shift work and SRH are consistent with other studies that concluded that shift work is not significantly related to nurses SRH [46,47]. In our study shift work, years of working, and department of employment were associated with SRH in the univariate analysis, but the multivariate analysis did not confirm an independent association on a statistically significant level. With regards to position and contrary to our results recent studies have shown that head nurses had elevated odds for low SRH, due to high job demands [27].

The principal limitation of our study was the lack of detailed information on non-respondents and therefore we were unable to assess if there was a subgroup that systematically failed to respond. An assessment of response bias was made comparing respondents and non-respondents with regard to gender and hospital of employment. Overall the respondents and non-respondents were quite similar according to gender but presented diversity according to hospital of employment. The highest non-response rate was observed at the General Hospital of Corfu. However, adjustment for this variable did not affect the results in the multivariate analysis. Furthermore the present data lack measurement of ill health, which according to most studies is one of the main domains considered when assessing health. In addition the present findings are based on cross sectional data, which preludes causal inference.

## Conclusion

Our study indicates that the health profile of nurses in Greece is relatively poor for this occupational group. In addition our study implies that some major health related behaviours are not considered directly by the group of nurses studied when self rating their health. This may have a negative impact on their ability to promote health in their patient population. Further understanding of nurses' beliefs and behaviours is required in order to develop policies and strategies that will enhance their health promotion role. This information could be used in targeted health education and health promotion efforts in this group of health professionals.

## Competing interests

The author(s) declare that they have no competing interests.

## Authors' contributions

NP had the idea for the project. ID designed the protocol and analyses with input from the other authors. NP organised the database for the analyses and YA run the analyses with input from NP. All authors interpreted the results. The manuscript was first drafted by NP and was revised and critically commented by all authors. ID is the guarantor of the paper.

## Pre-publication history

The pre-publication history for this paper can be accessed here:



## References

[B1] Idler EL, Benyamini Y (1997). Self rated health and mortality: a review of twenty-seven community studies. J Health Soc Behav.

[B2] Benyamini Y, Idler EL (1992). Community studies reporting associations between self rated health and mortality. Res Aging.

[B3] Mossey JM, Shapiro E (1982). Self rated health: a predictor of mortality among the elderly. Am J Public Health.

[B4] Benyamini Y, Idler EL, Leventhal H, Leventhal EA (2000). Positive affect and function as influences on self-assessments of health: expanding our view beyond illness and disability. J Gerontol B Psychol Sci Soc Sci.

[B5] Carlson P (2001). Risk behaviours and self rated health in Russia. J Epidemiol Community Health.

[B6] Fylkesnes K, Forde OH (1992). Determinants and dimensions involved in self evaluations of health. Soc Sci Med.

[B7] Manor O, Matthews S, Power C (2001). Self rated health and limiting longstanding illness: inter-relationships with morbidity in early adulthood. Int J Epidemiol.

[B8] Stewart-Brown S (1998). Emotional wellbeing and its relation to health. BMJ.

[B9] Miilunpalo S, Vuori I, Oja P, Pasanen M, Urponen H (1997). Self-rated health status as a health measure: the predictive value of self-reported health status on the use of physician services and on mortality in the working-age population. J Clin Epidemiol.

[B10] Wolinsky FD, Johnson RJ (1991). The use of health services by older adults. J Gerontol.

[B11] Shulz R, Mittelmark M, Kronmal R, Polak JF, Hirsch CH, German P, Bookwala J (1994). Predictors of perceived health status in elderly men and women. The Cardiovascular Health Study. Journal of Aging and Health.

[B12] Manderbacka K, Lundberg O, Marikainen P (1999). Do risk factors and health behaviours contribute to self rating of health?. Soc Sci Med.

[B13] Nies AH, Van Staveren WA (2003). Relation of dietary quality, physical activity and smoking habits to 10-year changes in health status in older Europeans in the SENECA study. Am J Public Health.

[B14] Poikolainen K, Vartiainen E, Korhonen HJ (1996). Alcohol intake and subjective health. Am J Epidemiol.

[B15] Lamb KL, Roberts K, Brodle DA (1990). Self perceived health among sports participants and non-sports participants. Soc Sci Med.

[B16] Thorlindsson T, Vilhjalmsson R, Valgeirsson G (1990). Sport participation and perceived health status: a study of adolescents. Soc Sci Med.

[B17] Moum T (1992). Self assessed health among Norwegian adults. Soc Sci Med.

[B18] Eriksson I, Unden AL, Elofsson S (2001). Self rated health. Comparisons between three different measures. Results from a population study. Int J Epidemiol.

[B19] Krause NM, Jay GM (1994). What do global self rated health items measure?. Medical Care.

[B20] Jylha M, Leskinen E, Alanen E, Leskinen AL, Heikkinen E (1986). Self rated health and associated factors among men of different ages. J Gerontology.

[B21] Hope A, Kelleher CC, O'Connor M (1998). Lifestyle practices and the health promoting environment of hospital nurses. J Adv Nurs.

[B22] Cheng Y, Kawachi I, Coakley EH, Schwartz J, Colditz G (2000). Associations between psychosocial work characteristics and health functioning in American women: a prospective study. BMJ.

[B23] Niedhammer I, Chea M (2003). Psychosocial factors at work and self reported health: Comparative results of cross sectional and prospective analyses of the French GA cohort. Occup Environ Med.

[B24] Laschinger HK, Almost J, Purdy N, Kim J (2004). Predictors of nurse managers health in Canadian restructured healthcare settings. Can J Nurs Leadersh.

[B25] Arafa MA, Nazel MWA, Ibrahim NK, Attia A (2003). Predictors of psychological well-being of nurses in Alexandria, Egypt. Int J Nurs Pract.

[B26] Dermouti E, Baker AB, Friedhelm N, Schanfels WB (2000). A model of burnout and life satisfactions amongst nurses. J Adv Nurs.

[B27] Lindholm M, Dejin-Karlsson E, Ostergren PO, Uden G (2003). Nurse managers' professional networks, psychosocial resources and self rated health. J Adv Nurs.

[B28] Estryn-behar M, Kaminski M, Peigne E, Bonnet N, Vaichere E, Gozlan C, Azoulay S, Giorgi M (1990). Stress at work and mental health status among female hospital workers. Br J Ind Med.

[B29] Weinberg A, Creed F (2000). Stress and psychiatric disorder in healthcare professionals and hospital staff. Lancet.

[B30] Thomsen S, Arnetz B, Nolan P, Soares J, Dallender J (1999). Individual and organizational well-being in psychiatric nursing: a cross cultural study. J Adv Nurs.

[B31] Adams A, Hons BA, Bond S (2000). Hospital nurses' job satisfaction, individual and organizational characteristics. J Adv Nurs.

[B32] Lundberg O, Manderbacka K (1996). Assessing reliability of a measure of self- rated health. Scand J Soc Med.

[B33] Daniilidou NV, Gregory S, Kyriopoulos JH, Zarvas DJ (2004). Factors associated with self rated health in Greece. Eur J Publ Health.

[B34] Gijsbers van Wijk C, van Vliet K, Kolk A, Everaerd W (1991). Symptom sensitivity and sex differences in physical morbidity: a review of health surveys in the United States and The Netherlands. Women Health.

[B35] Power C, Matthews S, Manor O (1996). Inequalities in self rated health in the 1958 birth cohort: lifetime social circumstances or social mobility?. BMJ.

[B36] Callaghan P (1999). Health beliefs and their influence on United Kingdom nurses' health related behaviors. J Adv Nurs.

[B37] Andrea MS, Walter V, Elena B, Alfea F, Piersante S (2001). A comparison of smoking, habits, beliefs and attitudes among Tuscan student nurses in 1992–1999. Eur J Epidem.

[B38] UNITE study group (2004). A survey of coronary risk factors and B-type natriuretic peptide concentrations in cardiac nurses from Europe: do nurses still practice what they preach?. Eur J Cardiov Nurs.

[B39] Hughes AM, Rissel C (1999). Smoking: rates and attitudes among nursing staff in central Sydney. Int J Nurs Practice.

[B40] Kaplan B, Yogev Y, Fisher M, Gall B, Dekel A, Sulkes J, Rabinerson D (2002). Self rated health attitudes and practices of obstetrics and gynaecology nurses in Israel. Clin Exp Obstet Gyn.

[B41] McKenna H, Slater P, McCance T, Bunting B, Spiers A, McElwee G (2003). The role of stress, peer influence and education levels on the smoking behaviour of nurses. Int J Nurs Studies.

[B42] Pratt JP, Overfield T, Hilton HG (1994). Health behaviors of nurses and general population women. Health Values.

[B43] Krassas GE, Tzotzas T, Tsametis C, Konstantinidis T (2001). Prevalence and trends in overweight and obesity among children and adolescents in Thessaloniki, Greece. J Pediatr Endocrinol Metab.

[B44] Gikas A, Sotiropoulos A, Panagiotakos D, Peppas T, Skliros E, Pappas S (2004). Prevalence, and associated risk factors, or self reported diabetes mellitus in a sample of Urban population in Greece: MEDICAL Exit Poll Research in Salamis. BMC Public Health.

[B45] Callaghan P, Fun MK, Yee FC (1997). Hong Kong nurses' health-related behaviours: implications for nurses' role in health promotion. J Adv Nurs.

[B46] Edell-Gustafsson U, Kritz KE, Bogren IK (2002). Self reported sleep quality, strain and health in relation to perceived working conditions in females. Scand J Caring Sci.

[B47] Skipper J, Jung FD, Coffey LC (1990). Nurses and shiftwork: effects on physical health and mental depression. J Adv Nurs.

[B48] Mamalakis G, Kafatos A (1996). Prevalence of Obesity in Greece. Int J Obes Relat Metab Disord.

